# Growth and differentiation factor 15 and NF‐κB expression in benign prostatic biopsies and risk of subsequent prostate cancer detection

**DOI:** 10.1002/cam4.3850

**Published:** 2021-03-30

**Authors:** Benjamin A. Rybicki, Sudha M. Sadasivan, Yalei Chen, Oleksandr Kravtsov, Watchareepohn Palangmonthip, Kanika Arora, Nilesh S. Gupta, Sean Williamson, Kevin Bobbitt, Dhananjay A. Chitale, Deliang Tang, Andrew G. Rundle, Kenneth A. Iczkowski

**Affiliations:** ^1^ Department of Public Health Sciences Henry Ford Hospital Detroit MI USA; ^2^ Medical College of Wisconsin Pathology, Milwaukee WI USA; ^3^ Department of Pathology Faculty of Medicine Chiang Mai University Chiang Mai Thailand; ^4^ Department of Pathology Henry Ford Hospital Detroit MI USA; ^5^ Department of Environmental Health Sciences Mailman School of Public Health Columbia University New York NY USA; ^6^ Department of Epidemiology Mailman School of Public Health Columbia University New York NY USA

**Keywords:** African Americans, cytokine, immunohistochemistry, inflammation, odds ratio

## Abstract

Growth and differentiation factor 15 (GDF‐15), also known as macrophage inhibitory cytokine 1 (MIC‐1), may act as both a tumor suppressor and promotor and, by regulating NF‐κB and macrophage signaling, promote early prostate carcinogenesis. To determine whether expression of these two inflammation‐related proteins affect prostate cancer susceptibility, dual immunostaining of benign prostate biopsies for GDF‐15 and NF‐κB was done in a study of 503 case‐control pairs matched on date, age, and race, nested within a historical cohort of 10,478 men. GDF‐15 and NF‐κB expression levels were positively correlated (*r* = 0.39; *p* < 0.0001), and both were significantly lower in African American (AA) compared with White men. In adjusted models that included both markers, the odds ratio (OR) for NF‐κB expression was statistically significant, OR =0.87; *p* = 0.03; 95% confidence interval (CI) =0.77–0.99, while GDF‐15 expression was associated with a nominally increased risk, OR =1.06; *p* = 0.27; 95% CI =0.96–1.17. When modeling expression levels by quartiles, the highest quartile of NF‐κB expression was associated with almost a fifty percent reduction in prostate cancer risk (OR =0.51; *p* = 0.03; 95% CI =0.29–0.92). In stratified models, NF‐κB had the strongest negative association with prostate cancer in non‐aggressive cases (*p* = 0.03), older men (*p* = 0.03), and in case‐control pairs with longer follow‐up (*p* = 0.02). Risk associated with GDF‐15 expression was best fit using nonlinear regression modeling where both first (*p* = 0.02) and second (*p* = 0.03) order GDF‐15 risk terms were associated with significantly increased risk. This modeling approach also revealed significantly increased risk associated with GDF‐15 expression for subsamples defined by AA race, aggressive disease, younger age, and in case‐control pairs with longer follow‐up. Therefore, although positively correlated in benign prostatic biopsies, NF‐κB and GDF‐15 expression appear to exert opposite effects on risk of prostate tumor development.

## Graphical abstract

In benign prostate biopsies we found expression of GDF‐15 and NF‐κB were positively correlated and lower in African American compared with white men. Prostate cancer risk estimates, however, suggest that NF‐κB and GDF‐15 expression exert opposite effects on prostate tumor development.

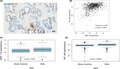

## 1 INTRODUCTION

Chronic inflammation is thought to increase cancer risk,^1^ and multiple lines of evidence support a link between chronic prostatic inflammation and prostate cancer development.^2^ Most prostate surgical specimens show histologic signs of inflammation,^3^, ^4^ and several studies from the Prostate Prevention Trial Cohort suggest prostatic inflammation increases prostate cancer risk.^4^, ^5^ However, a growing body of evidence from men with benign biopsies followed for subsequent prostate cancer suggests that histologic inflammation may decrease prostate cancer risk.^6^, ^7^, ^8^, ^9^ Although several studies have investigated associations between multiple circulating inflammatory markers and prostate cancer risk,^10^, ^11^, ^12^ no previous study has, to our knowledge, evaluated the association between pre‐diagnostic histologic markers of inflammation in the prostate and subsequent risk of cancer.

The inflammatory prostate tumor microenvironment includes leukocytes, cytokines, and complement components, all orchestrated by transcription factors. Nuclear factor kappa‐light‐chain‐enhancer of activated B cells, NF‐κB, is a transcription factor that regulates pro‐inflammatory gene expression and is constitutively activated in castration‐resistant prostate cancer.^13^ Prostatic epithelial cells may play a significant role in sustaining and amplifying inflammation through NF‐κB activation and local production of proinflammatory cytokines that result in the recruitment and activation of additional immune cells in the prostate.^14^ In the benign prostate, NF‐κB expression has been shown to be positively correlated with presence of histologic inflammation but not inflammation grade.^15^ The NF‐κB protein consists of homodimers and heterodimers formed from five subunits (p65, c‐rel, RelB, p50, and p52). In the canonical pathway, the inactive form of the p65/p50 dimer is associated with the inhibitor IκB and is retained in the cytoplasm.^16^ Once phosphorylated, IκB releases the p65/p50 dimer which then translocates to the nucleus activating the NF‐κB signaling pathways that can result in the progression of prostate cancer.^17^ In the pre‐malignant prostate, expression of the NF‐κB p65 subunit is more prominent in prostates with benign hyperplasia and expressed primarily in basal cells and cytoplasm.^18^, ^19^

Growth differentiation factor 15 (GDF‐15), also called prostate‐derived factor or PDF, NAG‐1, or MIC‐1, is a stress‐induced anti‐inflammatory cytokine expressed in macrophages, vascular smooth muscle cells, cardiomyocytes, adipocytes, and endothelial cells.^20^ GDF‐15 is not only expressed in prostate cancer but also in the prostatic pre‐malignant inflammatory environment.^21^ GDF‐15 possesses immunomodulatory functions^22^ and appears to have a key role in regulating inflammatory pathways in prostate, exhibiting both tumor‐suppressing and tumor‐promoting functions.^23^ GDF‐15 may suppress the activity of NF‐κB, indicating a tumor‐suppressing quality, but has also been shown to preferentially inhibit M1 macrophage formation, indicating a pro‐tumorigenic quality.^24^ Conversely, recent evidence from in vitro and in vivo pancreatic cancer models suggests that NF‐κB regulates GDF‐15, which in turn signals macrophages to suppress their proapoptotic activity thereby stimulating early cancer development.^25^ Data from prostate cancer immunohistochemical analyses indicate that NF‐κB and GDF‐15 are overexpressed in prostate tumor as compared to benign adjacent and normal prostatic tissues and overexpressed in bone metastasis specimens from patients.^26^

Given the complexity of the immune system network and the multidimensionality of host‐tumor interactions, NF‐κB and GDF‐15 may be signature molecules that can elucidate the molecular kinetics of inflammatory responses early in prostatic carcinogenesis. To evaluate the role of NF‐κB and GDF‐15 in early prostate cancer development, we studied the prostate cancer risk associated with the expression of these two molecules in benign prostatic biopsies from a matched case‐control sample nested within a large retrospective cohort of men with a benign biopsy testing models stratified by race and other case characteristics.

## 2 MATERIALS AND METHODS

### 2.1 Study sample

After Institutional Review Board approval, we identified a historical cohort of 10,478 men with a benign specimen collected by needle core biopsy of the prostate between January 1990 and December 2012. Within this cohort, a nested case‐control sample was assembled. Eligibility criteria included a recorded PSA level within a year of cohort entry, no history of a previous prostate cancer diagnosis, and that the surgical specimens were all biopsies and excluded transurethral resections. “Date of cohort entry” was defined as the date of initial benign prostatic biopsy; “date of case diagnosis” was the date of first cancer‐positive tissue specimen or the date a clinician first reported a clinical diagnosis of prostate cancer. Patients diagnosed with prostate cancer less than 1 year from date of initial biopsy were ineligible for the study. Within the cohort, we identified 725 potentially eligible cases diagnosed with prostate cancer prior to December 2012.

Incidence density sampling was used to select matched controls with replacement from all cohort members at risk at the time of case occurrence. Controls were randomly selected from among those cohort members who were free of prostate cancer at a follow‐up duration greater than or equal to the time between cohort entry and diagnosis of the matched case. Matching criteria included age at entry into cohort (±2 years), date of entry into cohort (± 2 years), and African American (AA) or White race. We were able to match 673 case‐control pairs with prostate biopsy tissue available for assaying. The remaining 52 cases could not be matched to form an eligible case/control pair due to (1) pathologic reexamination of recut “benign” biopsy at the time of the cohort entry detected malignancy (*n* = 19, 36.5%); (2) medical record review found prior history of prostate (*n* = 5, 9.6%) or other types of cancer (*n* = 4, 7.7%); (3) no eligible controls could be matched to a case (*n* = 14, 26.9%); (4) lack of analyzable prostate tissue (*n* = 8, 15.4%); and (5) unable to confirm incident prostate cancer diagnosis (*n* = 2, 3.8%).

### 2.2 Pathologic review

All benign tissue specimens were screened for the presence of cancer and inflammation by a genitourinary pathologist (NG or SW) blinded to disease progression. For cases with varying intensity of inflammatory infiltrate, the highest grade was recorded. The extent of inflammation was scored as focal (<10% of specimen), multifocal (10%–50% of specimen), or diffuse (>50% of specimen). Specimens with evidence of nonreported malignancy were reviewed by a second pathologist (NG or SW) before exclusion from the sample. Acute and chronic inflammation were evaluated by distribution of polymorphonuclear (PMN) and mononuclear cells (MNCs), respectively,^27^ with inflammation for each specimen scored by grade (mild, moderate, severe) and extent (focal: <10% of specimen; multifocal: 10%–50% of specimen; or diffuse: >50% of specimen) for both cell types.^28^ Figure S1 shows representative images of acute inflammation and different grades of chronic inflammation.

### 2.3 Specimen processing and immunohistochemistry

Formalin‐fixed biopsy specimens embedded in paraffin blocks were procured from Henry Ford Hospital biorepository. We randomly selected a subset of blocks for each case from blocks with available tissue cores. The number of biopsy cores analyzed ranged from one to 14 with 50% or more of the available cores examined for 80% of the study participants. For each block, serial sections at 5‐micron thickness were cut. The middle section was stained with hematoxylin and eosin and a pathologist (NG or SW) confirmed the tumor/benign status of the specimen and did histopathologic assessment of atrophy and inflammation. An unstained section from each specimen was used for double staining of NF‐κB and GDF‐15 using a modified immunohistochemistry (IHC) protocol. To control for variation in experimental conditions, matched case‐control pair biopsy specimens were processed on the same IHC run. Prostatectomy tissue was included as a positive control, and primary antibody was omitted as a negative control.

Slides were dried 30 min at 60°C, then deparaffinized down to deionized water. Antigen retrieval was performed on a PT Link (Dako) by preheating Target Retrieval Solution to 60°C and heating for 20 min at 97°C in pH =9 (Dako). Slides were washed with buffer for 5 min. Slides were stained on an automated Dako Autostainer (Agilent) with two drop zones at 150 µl each. Dual endogenous enzyme block was used (Dako), then protein block (Background Punisher, Biocare). Antibodies used were goat polyclonal antibody to GDF‐15 (catalog AF957, R&D Systems, Minneapolis) and rabbit monoclonal antibody to NF‐κB p65 (clone D14E12, Cell Signaling Technologies, Beverly, MA). Antibodies were applied as a GDF‐15 (1:900)/NF‐κB (1:1400) cocktail, for a 20‐min incubation. The secondary antibody was Mach2 double stain 2 (Biocare) micropolymers consisting of anti‐goat and anti‐rabbit conjugated to horseradish peroxidase and alkaline phosphatase respectively. Secondary antibodies were applied for 20 min followed by two buffer washes. Substrate chromogens were FLEX DAB for horseradish peroxidase and Ferangi Blue for alkaline phosphatase and applied for 10 min. Background was stained with hematoxylin Dako FLEX, and slides were rinsed with deionized water and oven dried 15 min. Cover slipping was with xylene‐substitute mounting medium EcoMount (Biocare).

### 2.4 Stained slide processing and image analysis

Whole slide scanning of NF‐κB and GDF‐15 stained matched benign biopsy slides was performed on two different platforms—Ventana iScan HT slide scanner (Roche Diagnostics) and/or Aperio CS2 Scanner (Leica Biosystems) at 40× magnification. All case‐control pairs were scanned for further staining analysis on the same platforms to avoid any bias due to different scanning modalities. The whole slide scanned image was further processed using the open source image analysis software QuPath v0.2.3.^29^ All glandular and periglandular regions immediately adjacent to the glands were annotated after excluding tissue regions with any tissue folding or other processing/staining artefacts. The annotated regions were then processed using a customized script developed to estimate the intensity and extent of NF‐κB and GDF‐15 expression. The tissue and blank areas were separated by overall staining optical density (OD) threshold of 0.08. The automated application separates positive staining (brown for GDF‐15 and blue for NF‐κB) from the background stain. Percentage of positive expression area (PPEA) was quantified as the number of positively stained pixels divided by the total number of tissue pixels, where the positive pixels are defined as pixels with brown staining OD above threshold of 0.15 for GDF‐15 and blue staining OD above threshold of 0.15 for NF‐κB. The average expression intensity (AEI) was calculated as the mean staining OD of positively stained pixels. The log transformed product of PPEA and AEI, log(PPEA*AEI), was used as the marker expression level of a subject.

### 2.5 Statistical analysis

Conditional logistic regression analyses were used to estimate odds ratios (ORs) for prostate cancer incidence during follow‐up. One‐to‐one matching controlled for age and race. Initial unmatched analyses examined the correlation between GDF‐15 and NF‐κB expression as well as the association of each marker with race and different histologic features. These unmatched analyses also included a covariate for IHC batch to control for experimental variation. Risk modeling of expression levels was done on both on a continuous and categorical scale—the latter as quartile groups. The thresholds for quartile groups were determined by the expression levels in control groups for AA and white samples separately. Initial univariate models assessed associations between prostate cancer incidence and marker expression level, overall and stratified by race. Next, multivariable models were fitted to jointly model GDF‐15 and NF‐κB expression while including variables for PSA level at benign biopsy and pathologist‐reviewed inflammation status. A series of multivariable models were fit stratified on race, case aggressive status, age, and time from cohort entry to case diagnosis (the latter two were stratified on the median). For each model, the statistical significance of the linear trend of quartile ORs was tested with a separate model where the quartile groupings were modeled as a single trend variable coded as 1 (lowest quartile) through 4 (highest quartile). A secondary analysis explored nonlinear associations between marker expression and prostate cancer risk. In this modeling approach, polynomials were used to represent nonlinear relationships that included testing higher order risk terms. The same covariates that were adjusted for in the primary analysis were included in these nonlinear models. A likelihood ratio *χ* ^2^ test was used to evaluate the significance of nested models.

## 3 RESULTS

### 3.1 Study sample

Among the eligible 673 case‐control pairs, we were able to successfully stain, scan, and image process NF‐κB and GDF‐15 stained slides for 508 case‐control pairs. An additional five pairs were removed because of incomplete covariate data or lack of prostate tissue on the IHC slide, resulting in a final analytic dataset of 503 case‐control pairs. Most pairs were excluded due to tissue loss during the multi‐stain process—a comparison of the cases in these excluded pairs (*n* = 170) versus those in the analytic dataset demonstrated little evidence of bias in terms of the cases that were selected for analysis with date of cohort entry the only variable that was significantly different between excluded cases and those in the analytic sample (Table S1). Excluded cases had biopsy specimens that were on average about a year older, which may explain why staining failed on these specimens. Table 1 shows the demographic and clinical characteristics of the analytic study sample that comprised 503 matched case‐control pairs. Cases were 46% AA with an average age of 64.5 years at cohort entry. Date of cohort entry ranged from 1990 to 2011; the median date of cohort entry was February 1997 for cases and March 1997 for controls. By design, cases were diagnosed with cancer at least 1‐year post‐cohort entry; the median time to cancer diagnosis was 6.34 years, with the longest interval from cohort entry to diagnosis being 18 years. Cases had a significantly higher PSA level at time of cohort entry but had a similar number of PSA tests between cohort entry and diagnosis (reference date for controls). Most cases were diagnosed with clinical stage 1 (74.3%) and 2 (23.5%) tumors. Approximately 26% of cases had advanced tumor grade, defined as either Gleason score 7 with a primary grade 4 (that is, International Society of Urological Pathology grade group 3), or higher. Both chronic and acute inflammation were 1–2 percentage points higher in controls compared to cases.

**TABLE 1:** Characteristics of analytic sample at baseline (503 matched pairs)

Variable	Response	Cases	Controls	*P* value
Race^a^	White	271 (54.0%)	271 (54.0%)	—
	African‐American	232 (46.0%)	232 (46.0%)	
Mean age at cohort entry (years)^a^ ± SD		64.5 ± 7.3	64.5 ± 7.3	—
Median date at cohort entry^a^		02/13/1997	03/17/1997	—
Median time to case diagnosis (years)^a^		6.34	—	—
Mean Serum PSA at cohort entry (ng/ml) ± SD		6.23 ± 0.28	5.42 ± 0.24	0.005
Mean number of PSA tests from cohort entry to diagnosis date ± SD		6.8 ± 0.2	7.2 ± 0.3	0.15
Prostatic Inflammation				
None		213 (42.3%)	197 (39.2%)	0.33
Chronic only		258 (51.3%)	263 (52.3%)	
Chronic and/or Acute		32 (6.4%)	43 (8.5%)	

Mean Serum PSA at time of case diagnosis (ng/ml) ± SD		24.6 ± 8.2	—	
Tumor stage^b^	1	374 (74.3%)	—	
	2	118 (23.5%)	—	
	3	7 (1.4%)	—	
	4	4 (0.8%)	—	
Gleason grade group^c^	1	232 (46.1%)	—	
	2	116 (23.1%)	—	
	3	47 (9.3%)	—	
	4	55 (10.9%)	—	
	5	32 (6.4%)	—	

Abbreviations: PSA, prostate specific antigen; SD, standard deviation.

^a^ Matching factor.

^b^ Clinical Stage unless missing, then Pathologic Stage reported.

^c^ Tumor grade based on prostatectomy and if missing, biopsy; 21 cases had missing tumor grade.

### 3.2 NF‐κB and GDF‐15 expression in benign prostate biopsies

**FIGURE 1:**
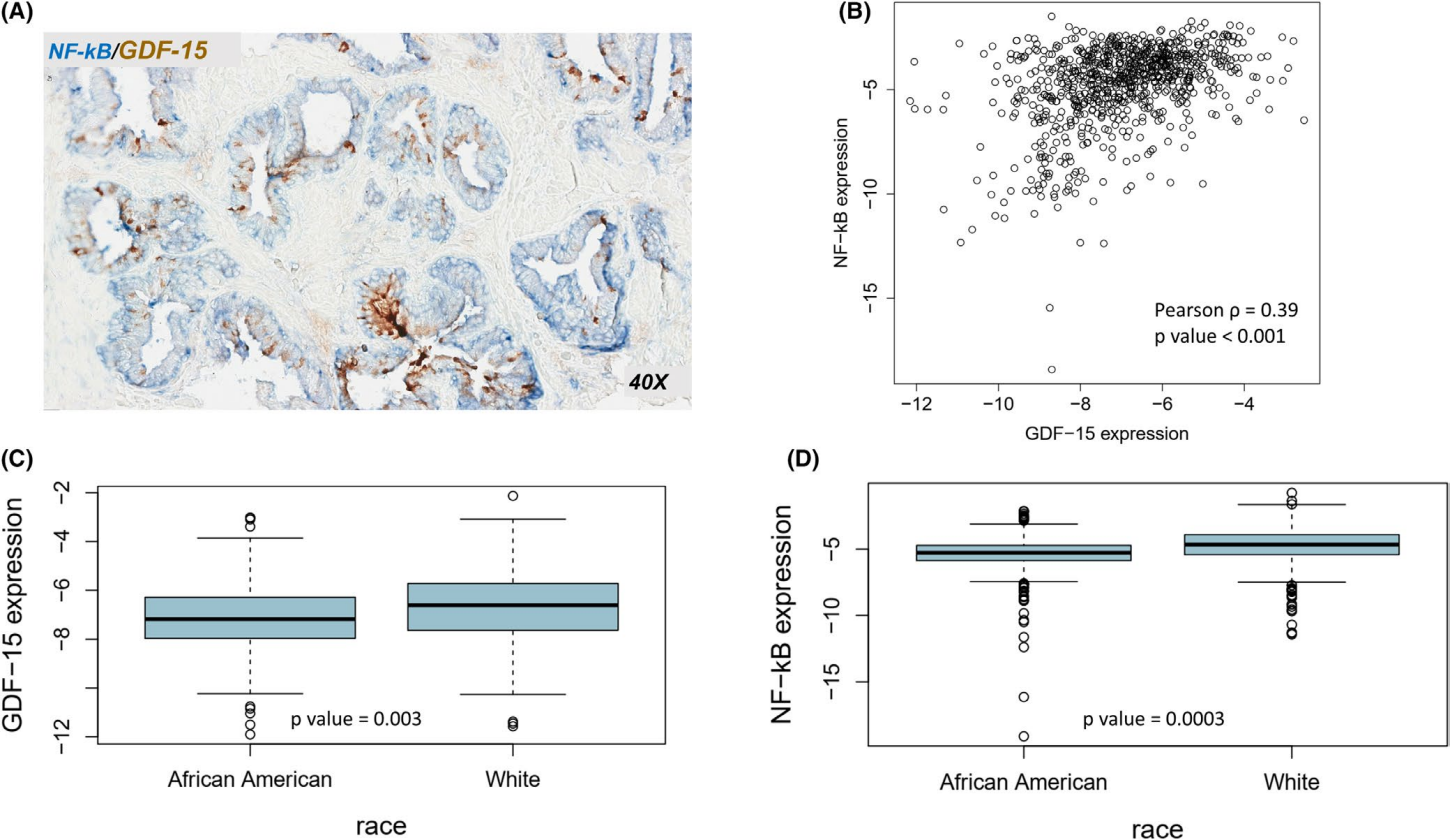
NF‐κB and GDF‐15 co‐expression. **(**A) Representative picture of NF‐κB (blue) and GDF‐15 (brown) staining in benign prostate glandular epithelium. (B) Scatter plot of NF‐κB and GDF‐15 expression levels in all prostate biopsy specimens (*n* = 820) and distribution by race of (C) NF‐κB and (d) GDF‐15 expression in prostate biopsy specimens (*n* = 450 Whites; *n* = 370 African Americans)

Some level of NF‐κB p65 expression was quantifiable in most benign prostatic biopsies, with the strongest expression observed primarily in the basal cells of prostate glandular epithelium (Figure 1A and Figure S3). GDF‐15 expression was also quantifiable in most biopsy specimens, however GDF‐15 expression tended to be more localized and generally restricted to the luminal aspect of glandular epithelium (Figure 1A and Figure S2). Isolated dark‐staining cells within the gland epithelium expressing GDF‐15 were also observed in a subset of the stained slides (Figure S2). When we analyzed the coexpression of NF‐κB and GDF‐15 in separately annotated regions, a strong positive correlation (*r* = 0.39; *p* < 0.0001) between the two markers emerged that was independent of race (Figure 1B). Expression of both GDF‐15 (Figure 1C) and NF‐κB (Figure 1D) was lower in AA men with racial differences in expression of both markers independent of case/control status. After adjusting for imaging platform and experimental batch, NF‐κB expression showed the strongest racial disparity (−5.41 ± 1.59 for AA vs. −4.79 ± 1.49 for White men; *p* = 0.0003), whereas for GDF‐15 expression the racial disparity in expression was less pronounced (−7.12 ± 1.40 for AA vs. −6.70 ± 1.38 for White men; *p* = 0.003).

**FIGURE 2:**
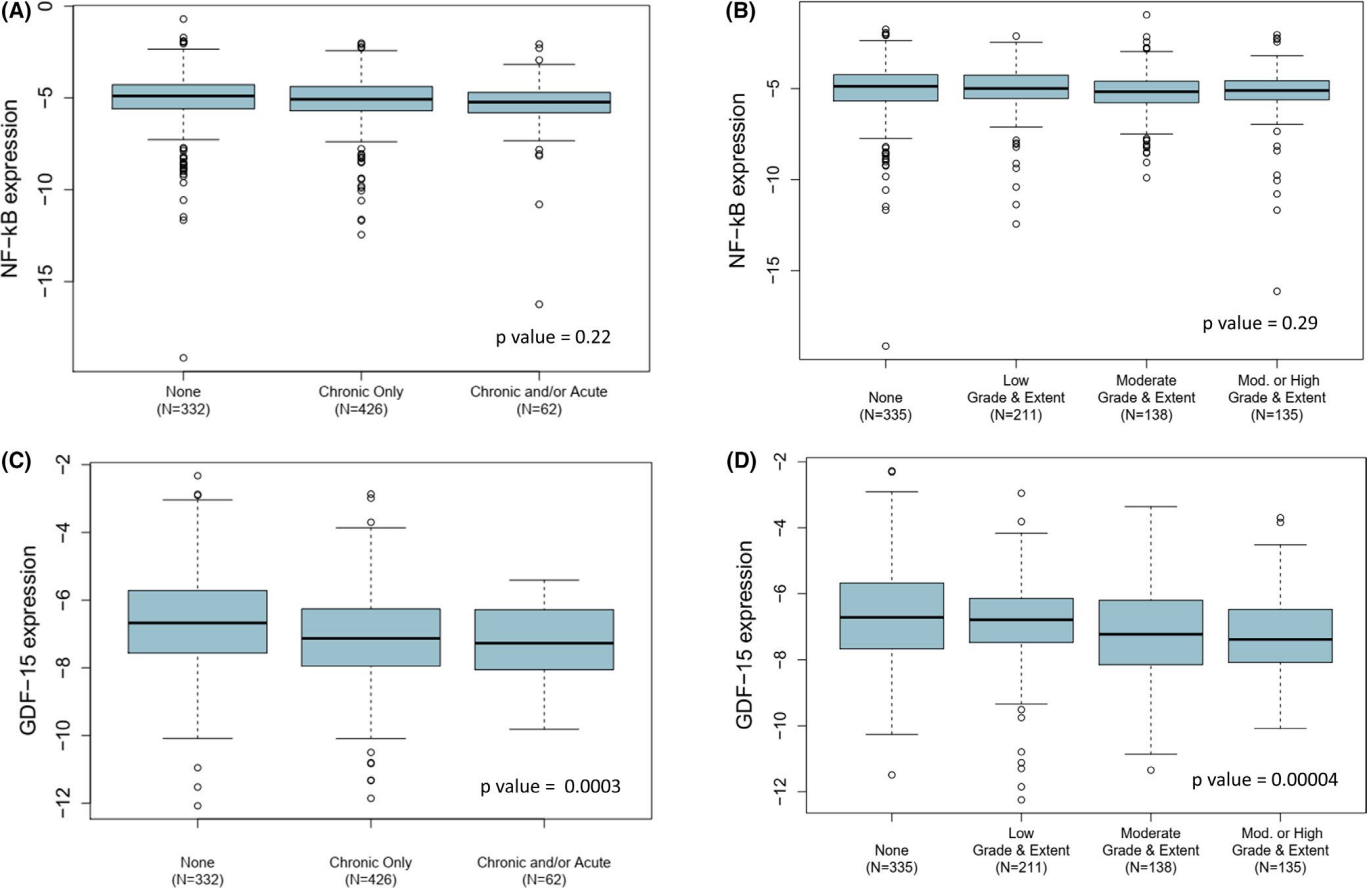
NF‐κB and GDF‐15 expression by inflammation status. **(**A) NF‐κB expression by chronic and acute inflammation status (*p* = 0.76); (B) GDF‐15 expression by chronic and acute inflammation status (*p* = 0.001); (C) NF‐κB expression by extent and grade of chronic inflammation (*p* = 0.62); (D) GDF‐15 expression by extent and grade of chronic inflammation (*p* < 0.0001)

Both NF‐κB and GDF‐15 expression levels were contrasted with prostatic inflammation (Figure 2), but only GDF‐15 expression significantly varied (Figure 2C,D). In prostatic biopsy specimens with chronic inflammation, GDF‐15 expression was significantly lower than in biopsies with no inflammation (*p* = 0.002). Examining the grade and extent of chronic inflammation, GDF‐15 expression showed an inverse linear trend with worsening inflammation (*p* = 0.00004). GDF‐15 expression was reduced by about five percent in specimens with at least a moderate grade or extent of inflammation compared to specimens with no inflammation.

### 3.3 NF‐κB and GDF‐15 expression and prostate cancer risk

Examination of NF‐κB and GDF‐15 expression levels by case‐control status showed slightly lower expression levels of NF‐κB in cases compared to controls (−4.81 ± 1.87 vs. −4.68 ± 1.80; *p* = 0.04), and a higher, albeit non‐significant, level of GDF‐15 expression in cases compared to controls. Case‐control differences in NF‐κB expression were most prominent in white men (−4.63 ± 1.80 vs. −4.42 ± 1.54; *p* = 0.03). When we modeled co‐expression of both markers, the negative prostate cancer risk association with NF‐κB expression remained (OR =0.87; 95% confidence interval [CI] =0.77–0.99; *p* = 0.03) and there was also a nominal positive association between case status and GDF‐15 expression (Table 2). A slightly stronger negative association of NF‐κB expression with case status was observed in White vs. AA men (OR =0.83 vs. 0.93) while the adjusted positive association of GDF‐15 expression was slightly higher in AA men (OR =1.09 vs. 1.03).

**TABLE 2:** Modeling of NF‐κB and GDF‐15 expression and prostate cancer risk in matched case‐control pairs

Variable	Unadjusted models		Adjusted models^a^	
	Odds ratio (95% CI)	*P* value	Odds ratio (95% CI)	*P* value
Full Sample (*n* = 503 pairs):				
NF‐κB	0.89 (0.79–1.00)	0.04	0.87 (0.77–0.99)	0.03
GDF−15	1.00 (0.94–1.12)	0.56	1.06 (0.96–1.17)	0.27
Whites (*n* = 271 pairs):				
NF‐κB	0.84 (0.71–0.99)	0.03	0.83 (0.70–0.98)	0.03
GDF−15	0.96 (0.85–1.08)	0.50	1.03 (0.90–1.17)	0.70
African Americans (*n* = 232 pairs):				
NF‐κB	0.94 (0.81–1.11)	0.47	0.93 (0.78–1.10)	0.39
GDF−15	1.06 (0.92–1.21)	0.44	1.09 (0.94–1.27)	0.24

Abbreviations: CI, confidence interval; *n*, number.

^a^ Adjusted models have covariates for PSA, inflammation, and both markers (NFκB and GDF15).

The expression of NF‐κB was next modeled in quartiles with the lowest expression level of each marker serving as the reference category (Table 3; see Figure S3 for representative staining by expression quartile). For the full sample, in the adjusted model the OR for the highest level of NF‐κB expression was 0.51 and nominally statistically significant (*p* = 0.03). Higher expression levels of GDF‐15 in the adjusted model showed a OR in the 4th quartile marginally higher than one but not statistically significant nor did the quartile ORs increase in a linear fashion. The inverse association of NF‐κB expression with prostate cancer was stronger and remained statistically significant in AA men.

**TABLE 3:** Modeling of NF‐κB and GDF‐15 expression levels and prostate cancer risk in matched case‐control pairs

Variable	Unadjusted models		Adjusted models^a^	
	Odds ratio (95% CI)	*P* value	Odds ratio (95% CI)	*P* value
Full sample (*n* = 503 pairs):				
NF‐κB				
2nd quartile	0.66 (0.44–0.99)	0.04	0.62 (0.40–0.94)	0.03
3rd quartile	0.72 (0.44–1.16)	0.17	0.70 (0.42–1.15)	0.16
4th quartile	0.53 (0.30–0.94)	0.03	0.51 (0.28–0.92)	0.03
Linear trend	0.83 (0.68–1.00)	0.04	0.81 (0.67–0.99)	0.04
GDF−15				
2nd quartile	0.77 (0.54–1.10)	0.16	0.83 (0.57–1.20)	0.31
3rd quartile	0.91 (0.64–1.29)	0.58	1.00 (0.69–1.45)	0.99
4th quartile	1.05 (0.72–1.51)	0.81	1.24 (0.83–1.83)	0.29
Linear trend	1.03 (0.92–1.16)	0.62	1.09 (0.96–1.23)	0.19
Whites (*n* = 271 pairs):				
NF‐κB				
2nd quartile	0.67 (0.39–1.13)	0.14	0.66 (0.38–1.14)	0.14
3rd quartile	0.80 (0.46–1.39)	0.43	0.80 (0.45–1.43)	0.45
4th quartile	0.61 (0.31–1.21)	0.16	0.62 (0.30–1.27)	0.19
Linear trend	0.88 (0.71–1.09)	0.25	0.88 (0.70–1.11)	0.29
GDF−15				
2nd quartile	0.70 (0.43–1.15)	0.16	0.75 (0.45–1.25)	0.27
3rd quartile	0.95 (0.59–1.54)	0.84	1.06 (0.63–1.77)	0.83
4th quartile	0.84 (0.52–1.36)	0.48	1.01 (0.60–1.70)	0.98
Linear Trend	0.98 (0.84–1.14)	0.79	1.04 (0.88–1.23)	0.64
African‐Americans (*n* = 232 pairs):				
NF‐κB				
2nd quartile	0.60 (0.31–1.16)	0.13	0.52 (0.26–1.04)	0.07
3rd quartile	0.48 (0.18–1.27)	0.14	0.42 (0.15–1.17)	0.10
4^th^ quartile	0.33 (0.11–1.02)	0.05	0.29 (0.09–0.96)	0.04
Linear trend	0.68 (0.47–0.99)	0.05	0.65 (0.44–0.97)	0.03
GDF−15				
2nd quartile	0.85 (0.51–1.44)	0.55	0.91 (0.53–1.58)	0.74
3rd quartile	0.85 (0.51–1.43)	0.54	0.89 (0.52–1.53)	0.67
th quartile	1.48 (0.82–2.65)	0.19	1.73 (0.92–3.25)	0.09
Linear trend	1.10 (0.92–1.32)	0.28	1.14 (0.95–1.38)	0.17

Abbreviation: CI, confidence interval.

^a^ Adjusted models have covariates for PSA, inflammation and both markers (NF‐κB and GDF‐15).

### 3.4 Stratified analyses

Table 4 shows stratified analyses by a case aggressive status, age and time between cohort entry and case diagnosis. Negative associations of NF‐κB expression with case status were more prominent for non‐aggressive cases—the OR for the highest (4th) expression quartile was 0.42 (95% CI =0.21–0.86) and the p for trend of the ORs was 0.03. For GDF‐15 expression, associations with prostate cancer risk did not appear to differ by disease aggressive status. Stratifying the case sample by age yielded the most disparate associations. Negative associations with NF‐κB expression and positive associations with GDF‐15 expression were observed only in older cases. In the highest quartile of NF‐κB expression, the OR for prostate cancer risk was 0.32; 95% CI =0.13–0.78; *p* = 0.01. In the highest quartile of GDF‐15 expression, the OR for prostate cancer risk was 1.77; 95% CI =1.00–3.12; *p* = 0.05. The linear trend of decreasing ORs for NF‐κB expression was statistically significant (*p* = 0.01). Stratifying by time to diagnosis revealed a slightly stronger negative association between NF‐κB expression and prostate cancer risk (OR for the highest quartile =0.33; 95% CI =0.13–0.80; *p* = 0.01) in the case‐control pairs with longer follow‐up. While GDF‐15 expression appeared to have a stronger association with prostate cancer risk in this stratum, the differences in effect estimates were not as striking. Analyses further stratified by race were also done for these three strata (Tables [Link], [Link], [Link]). Among less aggressive cases in AA men, NF‐κB and GDF‐15 inverse associations with prostate cancer were most stark—the OR for a linear trend in NF‐κB association was 0.57, whereas the comparable OR for GDF‐15 was 1.15 (Table S2). Associations of higher GDF‐15 expression with prostate cancer in older men (Table S3) was notably stronger in AA men (OR for the highest quartile =3.36; 95% CI =1.28– 8.82; *p* = 0.01). The negative association of NF‐κB expression with prostate cancer risk for cases with longer follow‐up (Table S4) was most prominent in white men (OR for the highest quartile =0.26; 95% CI =0.08–0.82; *p* = 0.03).

**TABLE 4:** Stratified models of NF‐κB and GDF‐15 expression levels and prostate cancer risk in matched case‐control pairs

Variables	Odds ratio (95% CI)	*P* value	Odds ratio (95% CI)	*P* value
Aggressive disease^a^	No (*n* = 325 pairs)		Yes (*n* = 178 pairs)	
NF‐κB				
2nd quartile	0.51 (0.31–0.84)	0.01	1.01 (0.43–2.38)	0.98
3rd quartile	0.70 (0.38–1.28)	0.25	0.79 (0.32–1.95)	0.61
4th quartile	0.42 (0.21–0.86)	0.02	0.76 (0.25–2.30)	0.63
Linear trend	0.77 (0.61–0.98)	0.03	0.90 (0.63–1.30)	0.58
GDF−15				
2nd quartile	0.86 (0.54–1.36)	0.52	0.74 (0.39–1.41)	0.36
3rd quartile	1.18 (0.74–1.89)	0.48	0.82 (0.45–1.51)	0.53
4th quartile	1.16 (0.71–1.88)	0.55	1.41 (0.71–2.82)	0.33
Linear trend	1.08 (0.93–1.26)	0.30	1.10 (0.89–1.37)	0.38
Age^b^	Young (*n* = 251 pairs)		Old (*n* = 252 pairs)	
NF‐κB				
2nd quartile	0.75 (0.41–1.38)	0.35	0.49 (0.26–0.92)	0.03
3rd quartile	0.70 (0.35–1.41)	0.32	0.70 (0.33–1.51)	0.37
4th quartile	0.79 (0.35–1.81)	0.58	0.32 (0.13–0.78)	0.01
Linear trend	0.92 (0.70–1.21)	0.54	0.72 (0.54–0.97)	0.03
GDF−15				
2nd quartile	0.47 (0.27–0.84)	0.01	1.30 (0.78–2.19)	0.32
3rd quartile	0.90 (0.52–1.56)	0.70	0.99 (0.58–1.68)	0.97
4th quartile	0.84 (0.47–1.51)	0.57	1.77 (1.00–3.12)	0.05
Linear trend	1.03 (0.86–1.24)	0.74	1.14 (0.96–1.36)	0.14
Time to Diagnosis^c^	Early (*n* = 252 pairs)		Late (*n* = 253 pairs)	
NF‐κB				
2nd quartile	0.58 (0.32–1.05)	0.07	0.63 (0.34–1.18)	0.15
3rd quartile	0.77 (0.38–1.53)	0.45	0.57 (0.27–1.20)	0.14
4th quartile	0.71 (0.31–1.61)	0.41	0.33 (0.13–0.80)	0.01
Linear trend	0.90 (0.69–1.18)	0.44	0.70 (0.52–0.94)	0.02
GDF−15				
2nd quartile	0.85 (0.51–1.41)	0.52	0.80 (0.46–1.39)	0.42
3rd quartile	1.01 (0.60–1.68)	0.98	0.97 (0.56–1.67)	0.90
4th quartile	0.94 (0.54–1.62)	0.82	1.53 (0.84–2.76)	0.16
Linear trend	1.00 (0.84–1.18)	0.98	1.17 (0.97–1.41)	0.09

Note:

All models have covariates for PSA, inflammation and both markers (NF‐κB and GDF‐15)

Abbreviation: CI, confidence interval.

^a^ Aggressive disease defined as Gleason group 3 or higher or PSA ≥20 or Tumor stage 3 or higher.

^b^ Young = <65 years old; old =65 years old or greater.

^c^ Early = less than 3.7 years; late =3.7 years or greater.

### 3.5 Nonlinear models

**FIGURE 3:**
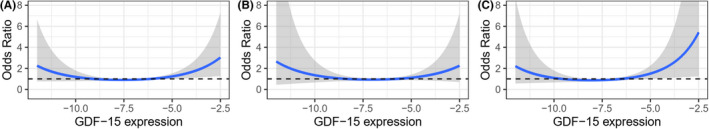
Nonlinear modeling of GDF‐15 expression and prostate cancer risk. Estimates for the following nonlinear model of prostate cancer risk: *β_0_*+ *PSA x β_1_* + *prostatic inflammation x β_2_* + *NF*‐*κB expression x β_3_* + *GDF*‐*15 expression x β_4_* + *(GDF*‐*15 expression)^2^ x β_5_* (PSA, prostatic inflammation and NF‐κB expression set at mean levels) for A) Full sample (*n* = 503 case‐control pairs); B) Whites (*n* = 271 case‐control pairs) and; C) African American (*n* = 232 case‐control pairs)

The modeling of NF‐κB and GDF‐15 risk by quartiles of expression suggested risk may increase in a nonlinear fashion; therefore, we tested a series of polynomial models that included higher order risk terms for both NF‐κB and GDF‐15 expression. Including covariables for prostate inflammation and PSA as well as the other marker, we first tested nested models with a second‐order and third‐order risk term for NF‐κB expression, but neither of these models significantly improved the fit over the simpler nested model. In a similar fashion, we tested the same nested models for GDF‐15 expression. Here, we found that the addition of a second order term for GDF‐15 expression significantly improved the fit of the model (*p* = 0.02); however, adding a third‐order term did not significantly improve the fit. Risk estimates for the model with the second‐order risk term over the range of GDF‐15 expression both for the full sample as well as the race‐stratified samples are depicted in Figure 3. In only AA men was a second‐order polynomial model a better fit to the GDF‐15 expression data (*p* = 0.04). Exploring further subsets of the data with this same nonlinear model, statistically significant first‐order and second‐order GDF‐15 risk estimates were found for subsamples defined by aggressive disease (*p* = 0.004), younger age (*p* = 0.01) and in case‐control pairs with longer follow‐up (*p* = 0.03) (Figure S4).

## 4 DISCUSSION

In a large, racially heterogeneous cohort of men with a benign prostatic biopsy, we observed suggestive associations of decreased NF‐κB and increased GDF‐15 expression in benign prostate with subsequent prostate cancer detection. These associations were more pronounced when stratifying case‐control pairs by age or case aggressive status. Opposite associations of NF‐κB and GDF‐15 expression with prostate cancer risk were observed despite the finding that expression of these two proteins positively correlated with each other. Overall, GDF‐15 expression was lower in AA benign prostate compared with benign prostate of white men which is consistent with the known effect of Vitamin D to upregulate GDF‐15,^30^ along with the tendency of AA men to have a relative Vitamin D deficiency. In a separate study focused on prostate tumor and adjacent benign tissue by Iczkowski et al.,^31^ our team found strong stage‐wise upregulation of GDF‐15 immunostaining in AA men. Since our analysis of benign biopsies shows a lower GDF‐15 expression in AA men, this implies an even greater upregulation from benign glands to intraprostatic tumor (pathologic stage 2) to extra‐prostatic tumor (higher stages), in which GDF‐15 is highest in AA men. Moreover, in benign biopsies, increased GDF‐15 expression had a suggestive association with prostate cancer in AA men, particularly when modeling GDF‐15 in a nonlinear fashion that allowed for a thresholding of GDF‐15 risk at the highest expression levels. This suggests a greater modulation of GDF‐15 may occur during carcinogenesis in AA men.

A previous study showed that in human prostate GDF‐15 suppresses the activity of NF‐κB, indicating a tumor‐suppressing quality, but it may also preferentially inhibit M1 macrophage formation, indicating a pro‐tumorigenic quality.^24^ During the course of prostatic carcinogenesis, M1 macrophages transform into a M2 phenotype, contributing to an immunosuppressive microenvironment that promotes tumor growth and metastasis.^32^ Recent evidence suggests that macrophages acquiring M2‐like characteristics may upregulate GDF‐15 expression in human cancer.^33^ In a study of men who developed prostate cancer and were subsequently followed for biochemical recurrence, we measured the co‐expression of M1/M2 macrophages and GDF‐15 in paired prostate specimens that reflected both the pre‐ and post‐malignant states.^34^ GDF‐15 expression showed a pattern in which the highest mean levels were observed in the normal prostatic glands adjacent to tumor. Moreover, men in whom the differential GDF‐15 expression was highest between tumor and normal adjacent glands had the lowest risk of biochemical recurrence. This suggests that while increased GDF‐15 expression may be a harbinger of prostate cancer development, overexpression of GDF‐15 may also serve a tumor suppressive function in terms of limiting the spread of cancer. Clearly, a high degree of variation in GDF‐15 expression exists in the different stages of prostate cancer,^21^ and studies of prostate cancer field cancerization ^35^, ^36^ suggest that GDF‐15 may be overexpressed in tumor‐adjacent normal glands, and this might explain the association we found between GDF‐15 expression and elevated prostate cancer risk in AA men.

NF‐κB can activate and regulate the expression of many inflammatory factors, which makes it the key promoter of the inflammatory response.^37^ Infection or hypoxia activates NF‐κB, which is inactive in cells, and activates inflammatory genes, induces the upregulation of cytokines, adhesion molecules, and vasoactive regulators and increases the concentration of further downstream cytokines such as tumor necrosis factor‐α (TNF‐α), interleukin‐6 (IL‐6), interleukin‐8 (IL‐8), and others.^38^ Similar to the few studies that have demonstrated NF‐κB expression in benign prostate,^15^, ^19^, ^39^ observable NF‐κB expression in our benign biopsy specimens was primarily cytoplasmic and in glandular basal cells suggesting that the NF‐κB expression detected was the inactive form of the enzyme. While our dual staining assay could possibly reduce the antigenicity of the monoclonal antibody to NF‐κB p65, given that the NF‐κB expression we were able to quantify in the majority of benign prostate specimens had a similar pattern of intensity and localization as has been previously reported in benign prostate ^15^, ^19^, ^39^ would suggest reduced antigenicity was not an issue. In prostate cancer, NF‐κB becomes constitutively activated in a high proportion of castration‐resistant prostate cancers,^13^, ^40^ but the active form of the enzyme is also detectable in early prostate carcinogenesis.^41^, ^42^

Previous studies suggested NF‐κB exerts a reciprocal interaction with GDF‐15.^24^, ^43^ Lambert et al. used a firefly luciferase construct to show that expression of the NF‐κB target, IL‐8, was downregulated by GDF‐15 in PC3 cells.^24^ GDF‐15 also inactivates NF‐κB signaling in dendritic cells, enabling induction of immune tolerance after heart transplantation.^43^ In our data, NF‐κB and GDF‐15 expression were positively correlated, and expression of GDF‐15 was negatively correlated with chronic inflammation levels. While these results contradict what has been observed in the tumor environment, both NF‐κB ^26^, ^39^, ^44^ and GDF‐15 ^26^, ^34^ expression levels are known to change as a result of malignant transformation in prostate and as we and others have previously shown, inflammation in benign prostate reduces the risk of subsequent cancer.^6^, ^7^, ^9^ Furthermore, the results of Ratnam et al., that show in pancreatic cancer NF‐κB is a direct regulator of GDF‐15, provide a possible mechanistic explanation for the inverse association of GDF‐15 and chronic inflammation in our data. In the former pancreatic cancer model, NF‐κB‐activated GDF‐15 suppresses macrophage cytotoxic activity. This model of early cancer immune surveillance could also occur in prostate cancer and may explain the correlation we observe between NF‐κB and GDF‐15 and the negative correlation of the latter with chronic inflammation.

NF‐κB expression levels are known to steadily increase as cancer progresses,^26^, ^39^, ^44^ but no prior study has examined the association of NF‐κB expression in benign prostate and subsequent prostate cancer risk. Others, including our group, have found racial differences in NF‐κB expression and prostate cancer aggressiveness.^45^ While we found NF‐κB expression was positively associated with tumor grade only in AA men,^31^ Hu et al.^45^ found the opposite albeit with a much smaller sample. Studies have consistently shown NF‐κB expression levels are associated with poor clinical outcome in prostate cancer patients,^17^, ^46^, ^47^, ^48^ but no reports of racial differences in these associations exist. In general, the ORs of the association of prostate cancer risk with NF‐κB expression were less than one and similar in magnitude for both races. In stratified analyses, however, AA men had greater disparity of NF‐κB expression associations with prostate cancer by disease aggressive status (NF‐κB decreased risk for nonaggressive disease but showed a suggestive positive association with aggressive disease) and age (NF‐κB decreased risk for older cases but showed a suggestive positive association with prostate cancer for younger cases). Among White men, NF‐κB expression appeared to have a stronger negative association with prostate cancer for cases that developed later in follow‐up.

The findings of our study may not be generalizable to all men. By virtue of having a prostate biopsy, cohort members were at elevated risk for prostate cancer and therefore cannot be considered representative of all men in the same age and race demographic. We excluded patients diagnosed with cancer within a year after cohort entry to minimize the chance of undetected prostate cancer at time of biopsy. Nonetheless, based on the age and risk profile of men in our cohort some likely had synchronous prostate cancer that was missed on biopsy. Despite the inherent shortcomings of a retrospective cohort design, embedding the cohort within a single health system permitted efficient sampling and complete incident case detection. Our nested case‐control sample also gave us the ability to estimate the prostate cancer risk associated with NF‐κB and GDF‐15 expression in benign prostate, which apart from unique studies such as the Prostate Cancer Prevention Trial,^49^ could not be carried out in a prospective setting.

In summary, NF‐κB and GDF‐15 expression appear to exert opposite effects on prostate tumor development, especially in AA men. The interplay of prostatic inflammation with NF‐κB and GDF‐15 expression in early prostate cancer development is unclear from our study results, although our findings suggest GDF‐15 may repress chronic inflammation in the benign prostate. There was no suggestion of a modifying effect of inflammation on prostate cancer risk associated with either NF‐κB or GDF‐15 expression. Much like the suspected diverse role of GDF‐15 in prostate carcinogenesis, NF‐κB may exert differing effects along the continuum of prostate tumor development. While our study sheds some light on the effect NF‐κB and GDF‐15 may have on prostate tumor development in the time just before malignant transformation, more modeling of the premalignant setting is needed to determine the dynamic changes in inflammatory mediators that initiate and support malignancy in prostate.

## Supporting information

Fig S1

Fig S2

Fig S3

Fig S4

Supplementary Material

Table S1‐S4

## Data Availability

The data used for this research are available upon request from the corresponding author.
