# Supplementary figures and images for: Growth and differentiation factor 15 and NF‐κB expression in benign prostatic biopsies and risk of subsequent prostate cancer detection
Source: Cancer Med. 2021 Mar 30;10(9):3013–25. doi: 10.1002/cam4.3850 (PMC8085972; doi:10.1002/cam4.3850)

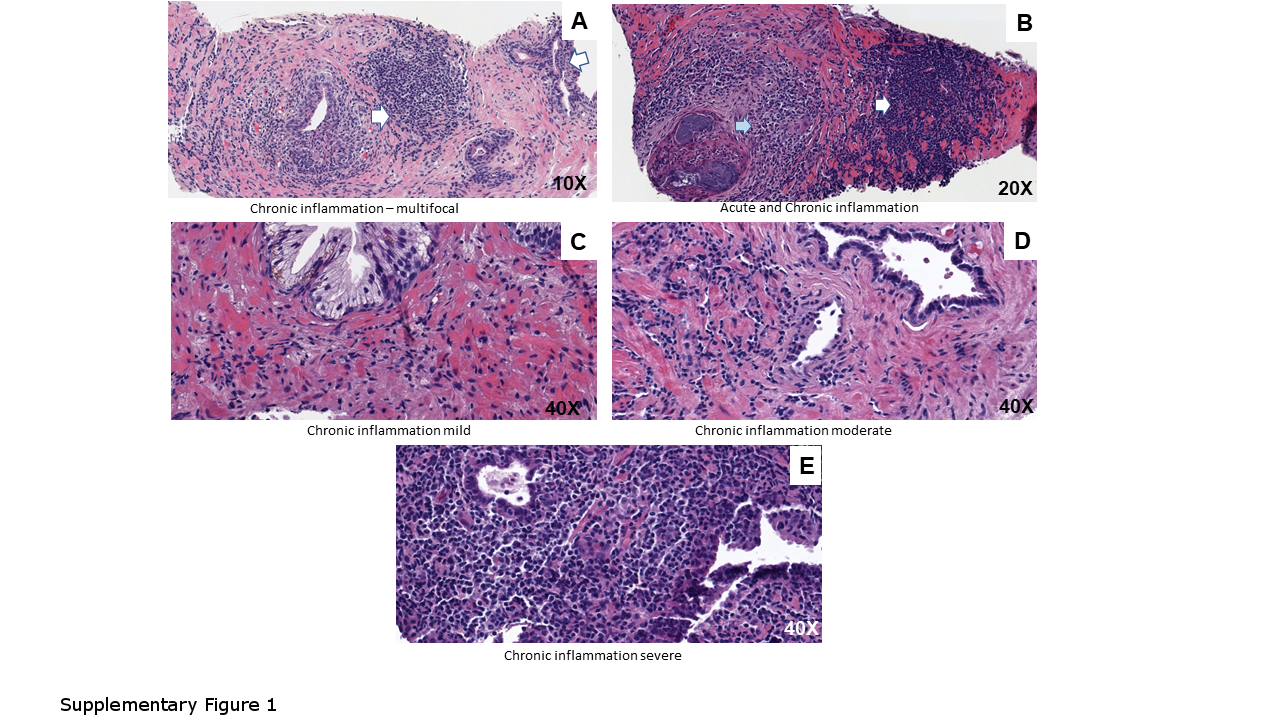

Supplement: Supplementary file 1 — Fig S1 [file CAM4-10-3013-s004.tif]

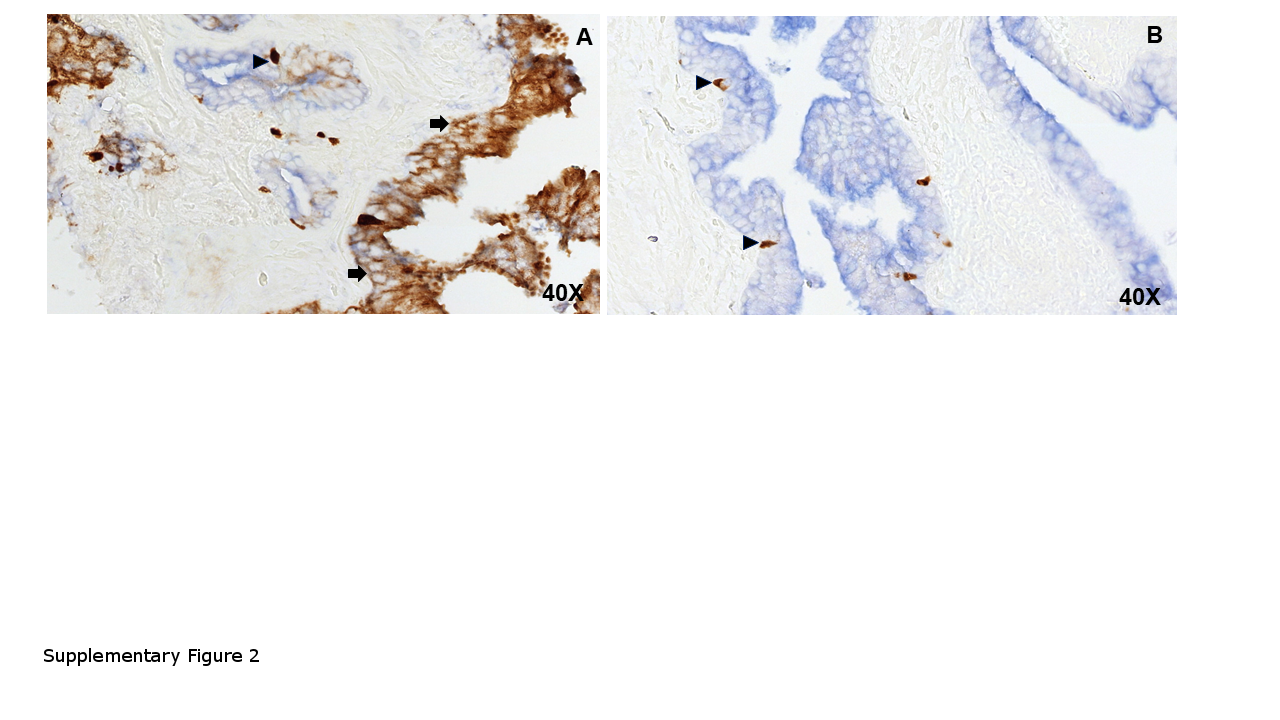

Supplement: Supplementary file 2 — Fig S2 [file CAM4-10-3013-s003.tif]

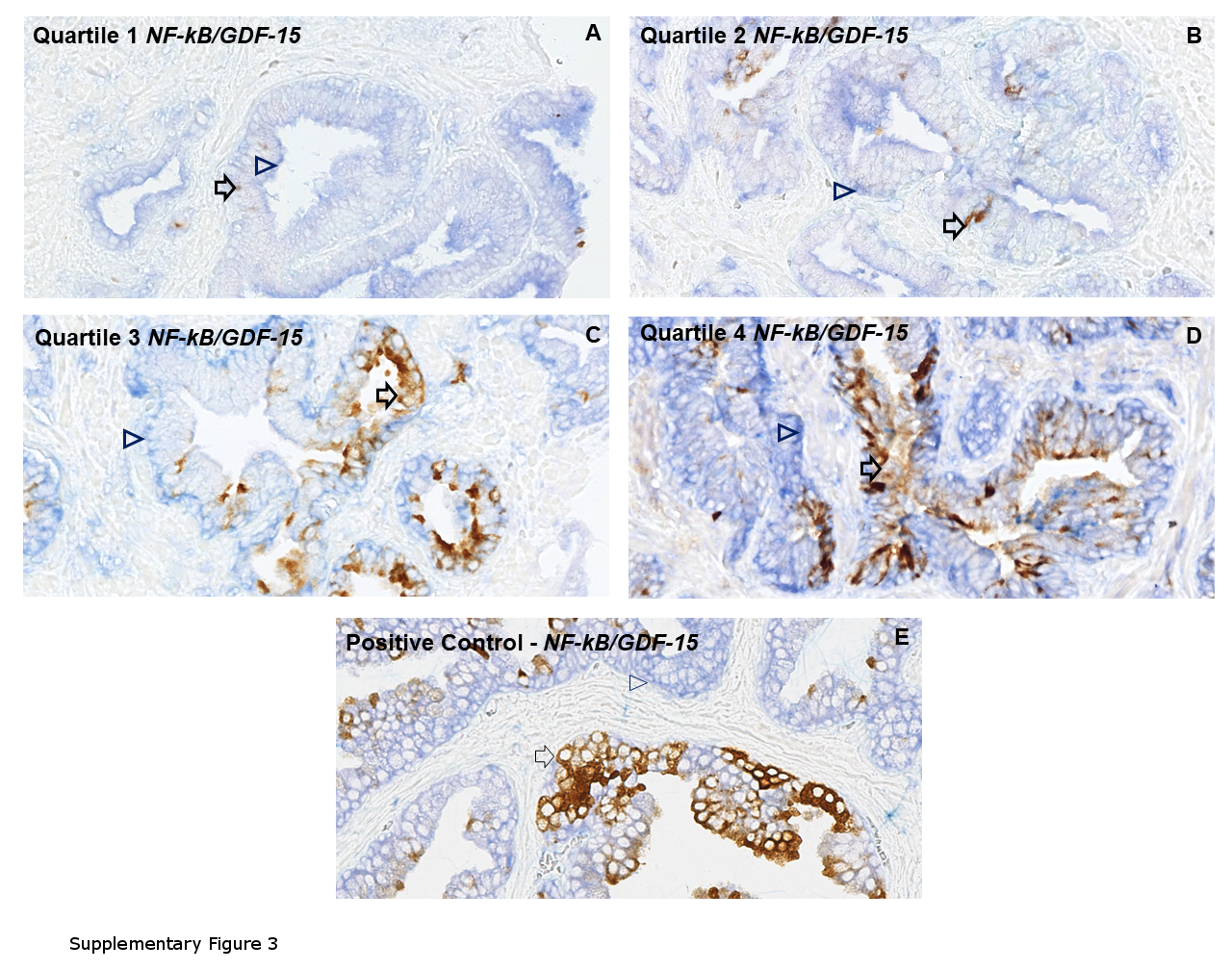

Supplement: Supplementary file 3 — Fig S3 [file CAM4-10-3013-s006.tif]

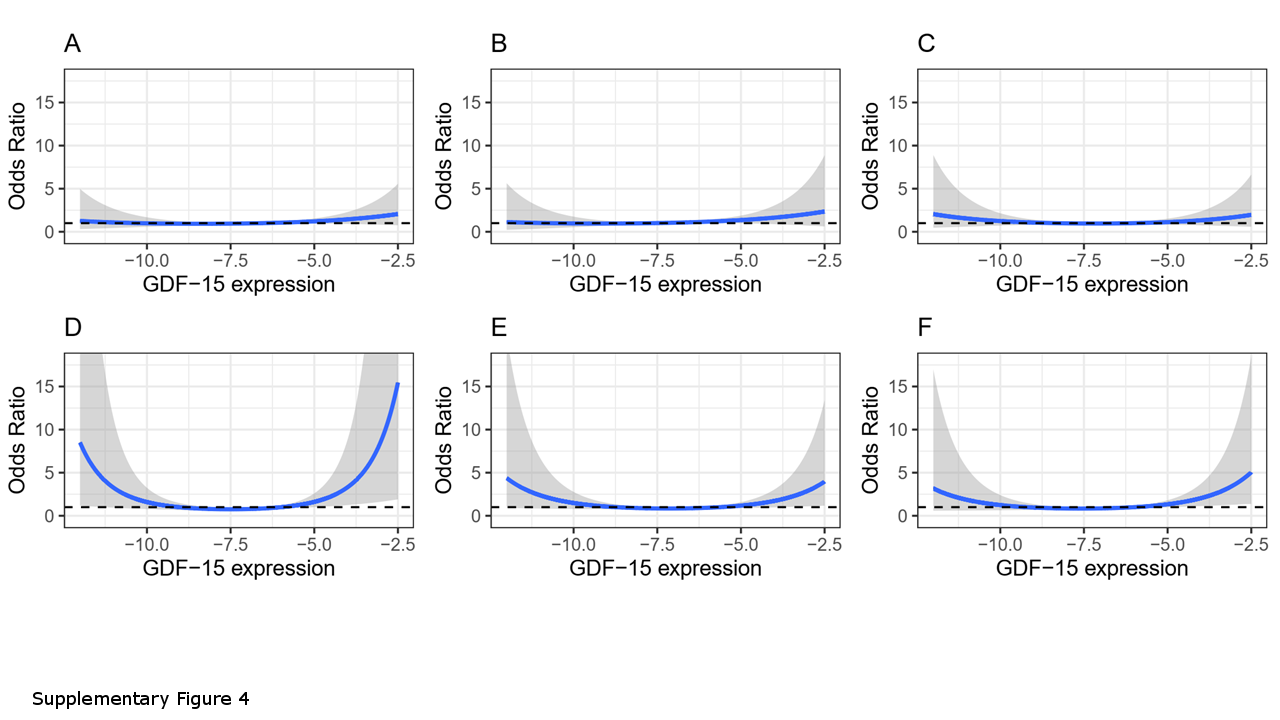

Supplement: Supplementary file 4 — Fig S4 [file CAM4-10-3013-s002.tif]
