# Supplementary material for: Growth and differentiation factor 15 and NF‐κB expression in benign prostatic biopsies and risk of subsequent prostate cancer detection
Source: Cancer Med. 2021 Mar 30;10(9):3013–25. doi: 10.1002/cam4.3850 (PMC8085972; doi:10.1002/cam4.3850)
Supplement: Supplementary file 5 — Supplementary Material [file CAM4-10-3013-s001.docx]

**Supplementary Figure Legends**

**Supplementary Figure 1.** Inflammation in the benign prostate biopsies. Representative images of (A) Chronic inflammation in multiple foci (white arrows), (B) Acute (blue arrow) and Chronic Inflammation (white arrow). Higher magnification representative images of areas of chronic inflammation of different severity (C) Mild with <100 MNC cells (D) Moderate with 100-500 MNC cells and, (E) Severe with >500 MNC cells.

**Supplementary Figure 2.** Representative images of GDF-15 expression (brown) in benign prostate biopsies. (A) GDF-15 expression was visible in the gland epithelium (arrows) and (B) in darker stained isolated cells in the gland (arrowheads). Note: NF-kB expression is shown in blue.

**Supplementary Figure 3.** Representative images of NF-kB and GDF-15 expression in different quartile groups used for analysis (A) Quartile group 1, (B) Quartile group 2, (C) Quartile group 3, (D) Quartile group 4. (E) positive control. All images are at 40X magnification depicting expression of NF-kB (arrowheads) and GDF-15 (arrows).

**Supplementary Figure 4. Nonlinear modeling of GDF-15 expression and prostate cancer risk.** Estimates for the following nonlinear model of prostate cancer risk: *β_0_+ PSA x β_1_ +* *prostatic inflammation x β_2_ + NF-κB expression x β_3_ + GDF-15 expression x β_4_ + (GDF-15 expression)^2^ x β_5_* (PSA, prostatic inflammation and NF-κB expression set at mean levels) for A) non-aggressive cases; B) older age (65 years or older); C) shorter follow-up (<3.7 years); D) aggressive cases (Gleason group 3 or higher or PSA ≥20 or Tumor stage 3 or higher); E) younger age (<65 years); F) longer follow-up (3.7 years or longer).
