# Supplementary material for: Growth and differentiation factor 15 and NF‐κB expression in benign prostatic biopsies and risk of subsequent prostate cancer detection
Source: Cancer Med. 2021 Mar 30;10(9):3013–25. doi: 10.1002/cam4.3850 (PMC8085972; doi:10.1002/cam4.3850)
Supplement: Supplementary file 6 — Table S1‐S4 [file CAM4-10-3013-s005.docx]

| **Supplemental Table 1. Comparison of cases included in analytic sample with excluded cases** | | | | |
| --- | --- | --- | --- | --- |
| **Variable** | **Response** | **Included Cases (n=503)** | **Excluded Cases (n=170)** | **p-value** |
|  |  |  |  |  |
| Race | White | 271 (53.9%) | 101 (59.4%) | 0.21 |
|  | African-American | 232 (46.1%) | 69 (40.6%) |  |
| Mean age at cohort entry (years) ± SD |  | 64.5 ± 7.3 | 65.5 ± 8.0 | 0.12 |
| Median date at cohort entry |  | 02/13/1997 | 03/16/1996 | 0.006 |
| Median time to case diagnosis (years) |  | 5.2 | 5.2 | 0.96 |
| Mean serum PSA at cohort entry (ng/ml) ± SD |  | 7.0 ± 0.3 | 8.1 ± 0.9 | 0.12 |
| Mean Serum PSA at time of case diagnosis (ng/ml) ± SD |  | 24.6 ± 8.2 | 32.7 ± 20.0 | 0.66 |
| Mean number of PSA tests from cohort entry to diagnosis date ± SD |  | 6.8 ± 0.2 | 6.5 ± 0.5 | 0.51 |
| Prostatic Inflammation |  |  |  |  |
| None |  | 214 (42.5%) | 71 (41.8%) | 0.95 |
| Chronic Only |  | 256 (50.9%) | 80 (47.1%) |  |
| Chronic and/or Acute |  | 32 (6.4%) | 10 (6.2%) |  |
| Tumor Clinical stage† | 1 | 374 (74.4%) | 125 (73.5%) | 0.85 |
|  | 2 | 118 (23.5%) | 40 (23.5%) |  |
|  | 3 | 7 (1.4%) | 4 (1.4%) |  |
|  | 4 | 4 (0.8%) | 1 (0.6%) |  |
| Biopsy Gleason grade group‡ | 1 | 232 (46.1%) | 71 (41.8%) | 0.63 |
|  | 2 | 116 (23.1%) | 39 (22.9%) |  |
|  | 3 | 47 (9.3%) | 19 (11.2%) |  |
|  | 4 | 55 (10.9%) | 24 (14.1%) |  |
|  | 5 | 32 (6.4%) | 9 (5.3%) |  |

† Clinical Stage unless missing, then Pathologic Stage reported

‡ Tumor grade based on prostatectomy and if missing, biopsy; 21 included cases and 8 excluded had missing tumor grade

Abbreviations: PSA – prostate specific antigen, SD – standard deviation

| **Supplemental Table 2: Modeling of** **NF-κB and GDF-15 expression levels**  **and Prostate Cancer Risk by Case Aggressive Status and race in matched case-control pairs** | | | | | | | | | | | | | |
| --- | --- | --- | --- | --- | --- | --- | --- | --- | --- | --- | --- | --- | --- |
|  | **Non-aggressive Cases** | | | | |  | **Aggressive Cases†** | | | | | | |
| **Variable** | **Odds Ratio**  **(95 % confidence interval)** | | | | **P value** |  | **Odds Ratio**  **(95 % confidence interval)** | | | | | | **P value** |
|  |  | | | |  |  |  | |  | |  | |  |
| Whites: | n=174 pairs | | | | |  | n=97 pairs | | | | | | |
| NF-κB |  | | | |  |  |  | | | | | |  |
| 2^nd^ quartile | 0.51 | (0.27 | - 0.99) | 0.05 | |  | 1.25 | (0.41 | | - 3.82) | | 0.70 | |
| 3^rd^ quartile | 0.86 | (0.41 | - 1.78) | 0.68 | |  | 0.70 | (0.25 | | - 1.97) | | 0.50 | |
| 4^th^ quartile | 0.48 | (0.20 | - 1.14) | 0.10 | |  | 0.98 | (0.25 | | - 3.91) | | 0.98 | |
| Linear trend | 0.85 | (0.64 | - 1.11) | 0.23 | |  | 0.91 | (0.59 | | - 1.41) | | 0.68 | |
| GDF-15 |  | | | |  |  |  | | | | | |  |
| 2^nd^ quartile | 0.78 | (0.40 | - 1.53) | 0.47 | |  | 0.71 | (0.30 | | - 1.65) | | 0.42 | |
| 3^rd^ quartile | 1.23 | (0.62 | - 2.45) | 0.55 | |  | 1.07 | (0.46 | | - 2.51) | | 0.87 | |
| 4^th^ quartile | 0.93 | (0.48 | - 1.80) | 0.83 | |  | 1.17 | (0.46 | | - 2.95) | | 0.74 | |
| Linear Trend | 1.03 | (0.84 | - 1.26) | 0.80 | |  | 1.08 | (0.80 | | - 1.46) | | 0.63 | |
|  |  | | | |  |  |  | |  | |  | |  |
| African-Americans: | n=151 pairs | | | | |  | n=81 pairs | | | | | | |
| NF-κB |  | | | |  |  |  | | | | | |  |
| 2^nd^ quartile | 0.45 | (0.20 | - 1.01) | 0.05 | |  | 0.71 | (0.15 | | - 3.44) | | 0.67 | |
| 3^rd^ quartile | 0.31 | (0.08 | - 1.11) | 0.07 | |  | 0.64 | (0.08 | | - 4.85) | | 0.66 | |
| 4^th^ quartile | 0.20 | (0.05 | - 0.88) | 0.03 | |  | 0.49 | (0.05 | | - 4.72) | | 0.54 | |
| Linear trend | 0.57 | (0.35 | - 0.92) | 0.02 | |  | 0.79 | (0.38 | | - 1.63) | | 0.52 | |
| GDF-15 |  | | | |  |  |  | | | | | |  |
| 2^nd^ quartile | 0.93 | (0.48 | - 1.78) | 0.82 | |  | 0.79 | (0.27 | | - 2.36) | | 0.68 | |
| 3^rd^ quartile | 1.07 | (0.54 | - 2.12) | 0.84 | |  | 0.54 | (0.20 | | - 1.46) | | 0.22 | |
| 4^th^ quartile | 1.55 | (0.73 | - 3.29) | 0.25 | |  | 2.42 | (0.68 | | - 8.63) | | 0.17 | |
| Linear trend | 1.15 | (0.91 | - 1.46) | 0.24 | |  | 1.08 | (0.78 | | - 1.50) | | 0.64 | |
| Note: all models have covariates for PSA, inflammation and both markers (NF-κB and GDF-15)  †Aggressive disease defined as Gleason group 3 or higher or PSA ≥20 or Tumor stage 3 or higher | | | | | | | | | | | | | |

| **Supplemental Table 3. Modeling of NF-κB and GDF-15 expression levels**  **and Prostate Cancer Risk by Age and Race in matched case-control pairs** | | | | | | | | | | | | | |
| --- | --- | --- | --- | --- | --- | --- | --- | --- | --- | --- | --- | --- | --- |
|  | **Young†** | | | | |  | **Old‡** | | | | | | |
| **Variable** | **Odds Ratio**  **(95 % confidence interval)** | | | | **P value** |  | **Odds Ratio**  **(95 % confidence interval)** | | | | | | **P value** |
|  |  | | | |  |  |  | |  | |  | |  |
| Whites: | n=143 pairs | | | | |  | n=128 pairs | | | | | | |
| NF-κB |  | | | |  |  |  | | | | | |  |
| 2^nd^ quartile | 0.60 | (0.27 | - 1.32) | 0.20 | |  | 0.71 | (0.31 | | - 1.61) | | 0.41 | |
| 3^rd^ quartile | 0.61 | (0.27 | - 1.40) | 0.25 | |  | 1.05 | (0.43 | | - 2.57) | | 0.91 | |
| 4^th^ quartile | 0.74 | (0.28 | - 1.95) | 0.55 | |  | 0.43 | (0.14 | | - 1.34) | | 0.15 | |
| Linear trend | 0.90 | (0.66 | - 1.23) | 0.50 | |  | 0.86 | (0.61 | | - 1.21) | | 0.39 | |
| GDF-15 |  | | | |  |  |  | | | | | |  |
| 2^nd^ quartile | 0.50 | (0.22 | - 1.10) | 0.08 | |  | 1.20 | (0.58 | | - 2.47) | | 0.62 | |
| 3^rd^ quartile | 1.19 | (0.53 | - 2.64) | 0.67 | |  | 0.93 | (0.46 | | - 1.90) | | 0.85 | |
| 4^th^ quartile | 0.86 | (0.39 | - 1.88) | 0.70 | |  | 1.27 | (0.60 | | - 2.70) | | 0.53 | |
| Linear Trend | 1.07 | (0.84 | - 1.35) | 0.59 | |  | 1.05 | (0.83 | | - 1.33) | | 0.71 | |
|  |  | | | |  |  |  | |  | |  | |  |
| African-Americans: | n=108 pairs | | | | |  | n=124 pairs | | | | | | |
| NF-κB |  | | | |  |  |  | | | | | |  |
| 2^nd^ quartile | 1.06 | (0.36 | - 3.14) | 0.92 | |  | 0.21 | (0.07 | | - 0.66) | | 0.01 | |
| 3^rd^ quartile | 0.74 | (0.16 | - 3.37) | 0.69 | |  | 0.26 | (0.05 | | - 1.31) | | 0.10 | |
| 4^th^ quartile | 0.72 | (0.12 | - 4.37) | 0.72 | |  | 0.17 | (0.03 | | - 0.97) | | 0.05 | |
| Linear trend | 0.90 | (0.50 | - 1.63) | 0.73 | |  | 0.52 | (0.30 | | - 0.91) | | 0.02 | |
| GDF-15 |  | | | |  |  |  | | | | | |  |
| 2^nd^ quartile | 0.43 | (0.18 | - 1.06) | 0.07 | |  | 1.81 | (0.81 | | - 4.06) | | 0.15 | |
| 3^rd^ quartile | 0.62 | (0.28 | - 1.38) | 0.24 | |  | 1.04 | (0.44 | | - 2.43) | | 0.93 | |
| 4^th^ quartile | 0.88 | (0.33 | - 2.32) | 0.80 | |  | 3.36 | (1.28 | | - 8.82) | | 0.01 | |
| Linear trend | 0.95 | (0.71 | - 1.28) | 0.75 | |  | 1.28 | (0.97 | | - 1.69) | | 0.08 | |
| Note: all models have covariates for PSA, inflammation and both markers (NF-κB and GDF-15)  †Young = <65 years old;  ‡Old = 65 years old or greater | | | | | | | | | | | | | |

| **Supplemental Table 4. Modeling of NF-κB and GDF-15 expression levels**  **and Prostate Cancer Risk by time between cohort entry and case diagnosis and race in matched case-control pairs** | | | | | | | | | | | | | |
| --- | --- | --- | --- | --- | --- | --- | --- | --- | --- | --- | --- | --- | --- |
|  | **Early†** | | | | |  | **Late‡** | | | | | | |
| **Variable** | **Odds Ratio**  **(95 % confidence interval)** | | | | **P value** |  | **Odds Ratio**  **(95 % confidence interval)** | | | | | | **P value** |
|  |  | | | |  |  |  | |  | |  | |  |
| Whites: | n=138 pairs | | | | |  | n=133 pairs | | | | | | |
| NF-κB |  | | | |  |  |  | | | | | |  |
| 2^nd^ quartile | 0.55 | (0.26 | - 1.14) | 0.11 | |  | 0.72 | (0.30 | | - 1.73) | | 0.46 | |
| 3^rd^ quartile | 0.93 | (0.42 | - 2.08) | 0.86 | |  | 0.59 | (0.24 | | - 1.47) | | 0.26 | |
| 4^th^ quartile | 1.27 | (0.47 | - 3.41) | 0.64 | |  | 0.26 | (0.08 | | - 0.82) | | 0.02 | |
| Linear trend | 1.06 | (0.78 | - 1.45) | 0.70 | |  | 0.67 | (0.46 | | - 0.96) | | 0.03 | |
| GDF-15 |  | | | |  |  |  | | | | | |  |
| 2^nd^ quartile | 0.61 | (0.30 | - 1.25) | 0.18 | |  | 0.85 | (0.38 | | - 1.88) | | 0.68 | |
| 3^rd^ quartile | 0.87 | (0.42 | - 1.81) | 0.72 | |  | 1.05 | (0.47 | | - 2.34) | | 0.90 | |
| 4^th^ quartile | 0.70 | (0.33 | - 1.49) | 0.35 | |  | 1.34 | (0.61 | | - 2.94) | | 0.46 | |
| Linear Trend | 0.93 | (0.73 | - 1.18) | 0.54 | |  | 1.14 | (0.89 | | - 1.46) | | 0.30 | |
|  |  | | | |  |  |  | |  | |  | |  |
| African-Americans: | n=113 pairs | | | | |  | n=119 pairs | | | | | | |
| NF-κB |  | | | |  |  |  | | | | | |  |
| 2^nd^ quartile | 0.53 | (0.17 | - 1.63) | 0.27 | |  | 0.55 | (0.22 | | - 1.39) | | 0.21 | |
| 3^rd^ quartile | 0.32 | (0.07 | - 1.48) | 0.15 | |  | 0.53 | (0.12 | | - 2.31) | | 0.40 | |
| 4^th^ quartile | 0.19 | (0.03 | - 1.09) | 0.06 | |  | 0.43 | (0.08 | | - 2.36) | | 0.33 | |
| Linear trend | 0.57 | (0.32 | - 1.03) | 0.06 | |  | 0.73 | (0.42 | | - 1.25) | | 0.25 | |
| GDF-15 |  | | | |  |  |  | | | | | |  |
| 2^nd^ quartile | 1.13 | (0.52 | - 2.45) | 0.75 | |  | 0.73 | (0.32 | | - 1.64) | | 0.44 | |
| 3^rd^ quartile | 1.11 | (0.51 | - 2.41) | 0.79 | |  | 0.74 | (0.33 | | - 1.67) | | 0.47 | |
| 4^th^ quartile | 1.22 | (0.52 | - 2.87) | 0.65 | |  | 2.18 | (0.81 | | - 5.83) | | 0.12 | |
| Linear trend | 1.06 | (0.82 | - 1.37) | 0.67 | |  | 1.22 | (0.91 | | - 1.63) | | 0.18 | |
| Note: all models have covariates for PSA, inflammation and both markers (NF-κB and GDF-15)  †Early = less than 3.7 years  ‡Late = 3.7 years or greater | | | | | | | | | | | | | |
